# Vector‐Driven Projection and Backprojection

**DOI:** 10.1002/mp.70581

**Published:** 2026-07-29

**Authors:** Ismail Melik Turker, Isa Yildirim

**Affiliations:** ^1^ Department of Electronics and Communication Engineering Istanbul Technical University Istanbul Türkiye

## Abstract

**Background:**

Projection and backprojection are fundamental operators in tomographic image reconstruction. Existing approaches—namely pixel‐driven (PD), ray‐driven (RD), and distance‐driven (DD)—are subject to well‐known trade‐offs among interpolation artifacts, geometric flexibility, and computational cost.

**Purpose:**

This study aims to introduce a unified framework that systematically models and resolves the intrinsic limitations of current projection topologies. Within this framework, a novel topology called vector‐driven (VD) is proposed to improve geometric flexibility without sacrificing computational efficiency.

**Methods:**

A three‐level hierarchical formulation—comprising topology, algorithm, and implementation—is also developed to isolate the structural origins of artifacts and performance bottlenecks in projection operators. The proposed VD projection and backprojection method represents all tomographic system components as vectors in 3D space and performs non‐orthogonal projections that preserve geometric symmetry between forward and backward operations.

**Results:**

Experimental validation was conducted using multiple geometric configurations, and quantitative image quality metrics (SSIM, RMSE, PSNR, NCC, UIQI) were compared against PD, RD, and DD baselines. VD produced artifact‐free output across all tested geometries and reached near‐perfect image‐quality scores matching the leading RD‐PD baseline combination. VD attained the shortest wall‐clock time in both directions, running about 37% faster than an optimised PD algorithm in forward projection and reaching near parity in backprojection, while remaining 31%–57% faster than De Man's DD and 62%–82% faster than Siddon's RD in both directions. This advantage reflects VD's balanced profile of low arithmetic workload of 13.7–24.0 GFLOP per call (giga floating‐point operations), high pipeline efficiency with 3.6–4.4 instructions per cycle (IPC), and low DRAM traffic of about 5 GB read per call.

**Conclusions:**

VD unifies forward and backward projection within a single vector‐based model and, in the configurations tested, performs competitively with established projection topologies in both accuracy and computational cost.

## 1 INTRODUCTION

Projection and backprojection are the core operations in tomographic image reconstruction. Projection simulates the formation of 2D X‐ray images from a 3D object, while backprojection redistributes those 2D projections into the 3D domain to reconstruct the original volume. They are essential in analytic methods, where backprojection is the key step, and indispensable in iterative reconstruction algorithms, where both forward and backward projections are applied at every iteration.

**FIGURE 1:**
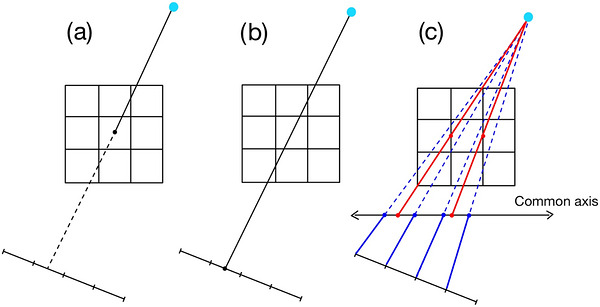
Illustration of the projection and backprojection topologies: (a) PD, (b) RD, and (c) DD.

Over the years, several projection topologies have been introduced to model the ray‐object‐detector interaction. Among the most well‐known are the *Pixel‐Driven* (PD), *Ray‐Driven* (RD), and *Distance‐Driven* (DD) topologies. In PD,^1^, ^2^, ^3^, ^4^ each voxel is projected onto the detector plane, and interpolation is applied using neighboring detector values. While PD works well in backprojection, it is prone to artifacts in forward projection. RD, in contrast, simulates rays emitted from the source to each detector cell and integrates voxel values along the ray path. It performs well for projection but introduces artifacts in backprojection. In both cases, these artifacts appear as Moiré‐like striping patterns. They arise from finite pointwise sampling — voxel centers in PD, rays in RD — which leaves intermediate elements on the receiving side (detector cells or voxels, respectively) without any contribution. Several algorithms such as those by Joseph,^5^ Siddon,^6^ Köhler,^7^ Chen et al.,^8^ and Gao^9^ have been proposed to improve its efficiency and accuracy. An overview of these topologies is illustrated in Figure 1, which highlights their conceptual differences in ray tracing and data sampling.

**FIGURE 2:**
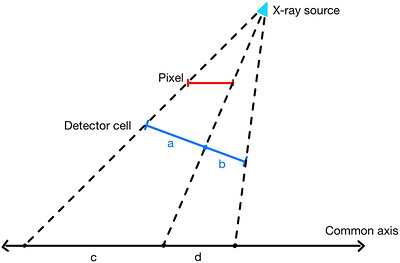
The figure illustrates the distortion of proportions caused by tilted detector problem. Specifically, $\frac{a}{\left(a+b\right)}\neq \frac{c}{\left(c+d\right)}$, because the detector extension is not parallel to the common axis and pixel extension.

DD was introduced by De Man et al.^10^, ^11^ as a hybrid approach that models both voxels and detector cells as rectangular regions and computes their overlap on a common plane. Although DD offers accurate and symmetric behavior for both projection and backprojection, its reliance on geometric alignment introduces major challenges under arbitrary rotations. Specifically, the topology suffers from several critical issues: the *Irregular Intersection Problem*, where distorted and non‐rectangular projections lead to inaccurate overlap calculations; the *Tilted Detector Problem*, where geometric distortions between the mapped detector and voxel boundaries degrade proportionality and spatial alignment (see Figure 2); and the *Index Matching Problem*, where irregular shapes hinder sequential memory access and lead to indexing mismatches during iteration. These problems limit the applicability of DD, and their severity has not been sufficiently investigated in the existing literature.

To improve the efficiency of DD‐based algorithms, Basu et al.^12^ proposed a branchless implementation that replaces conditional statements with arithmetic operations, thereby mitigating CPU branch misprediction penalties. Similarly, Miao et al.^13^ introduced the improved distance‐driven (IDD) method, which enhances depth‐wise accuracy by jointly processing the top and bottom boundaries of neighboring image slices. In addition, several studies have focused on accelerating both standard and branchless DD algorithms by leveraging GPU architectures.^14^, ^15^, ^16^ While these contributions improve algorithmic and implementation‐level efficiency, they do not address the geometric limitations inherent to the DD topology, particularly under complex detector orientations.

Another important method is the separable footprint (SF) approach introduced by Long et al.^17^ In this method, the voxel footprint on the detector is modeled using trapezoidal or rectangular functions, and the voxel's contribution is computed as separable horizontal and vertical footprints. Topologically, SF belongs to the PD family because both forward and backward projections are driven by voxels that are projected onto the detector plane, where the contribution is then computed. Unlike classical PD, which treats each voxel as a single point, SF distributes the voxel's contribution through a SF. This volumetric modeling effectively reduces artifacts in forward projection and improves accuracy. However, these benefits come at the cost of additional computational overhead and a more complex mathematical structure, making the implementation more challenging and reducing computational performance.

A related approach is the area integral model (AIM) proposed by Yu et al.,^18^ which computes voxel contributions as true surface integrals over the detector cell area. AIM can further improve geometric accuracy by directly integrating over the projected voxel area without intermediate approximations. Similar to SF, however, AIM incurs significant computational cost due to its mathematically intensive formulation, which can limit its practicality for large‐scale 3D imaging.

Despite this progress, current topologies face important limitations: PD and RD are inherently asymmetric and introduce artifacts in one direction; DD requires alignment between the object and detector planes, making it fragile under complex geometries. Methods like SF and AIM, while accurate, are computationally costly and do not generalise efficiently to arbitrary multi‐axis rotations, since deviations from canonical detector geometry strain their formulations and inflate computational complexity. Moreover, most studies in the literature conflate topology, algorithm, and implementation, making it difficult to isolate the source of limitations or evaluate proposed improvements consistently.

In this study, we first introduce a three‐level framework to systematically define the projection problem: *topology*, *algorithm*, and *implementation*. This framework enables a clearer conceptual understanding and guides the analysis of existing approaches. Based on this framework, we propose a novel projection topology called *vector‐driven* (VD), together with a forward and backward projection algorithm derived from vector algebra. VD can map both voxels and detector cells onto the corresponding plane via non‐orthogonal vector projections, with the same mathematical formulation applied in both directions. The forward and backward operators are therefore symmetric and produce artifact‐free output, avoiding the directional striping that characterises PD in forward projection and RD in backward projection. Because the topology operates on relative spatial positions rather than fixed coordinate alignments, it remains robust under arbitrary detector orientations and rotation axes, removing the alignment requirements that constrain DD and SF. At the algorithm level, the operator reduces to compact vector primitives such as dot and cross products, which yield a light arithmetic workload and decompose naturally into small independent units amenable to parallel execution on modern multi‐core hardware.

The proposed method is evaluated both in terms of accuracy and computational performance and compared with existing PD, RD, and DD topologies. Experimental results demonstrate that VD achieves better performance in both projection and backprojection under complex geometrical conditions while significantly reducing computation time.

## 2 METHODS

### 2.1 Problem definition

In computed tomography, the physical measurement at a detector cell corresponds to the attenuation of X‐rays as they travel through an object. For a single ray indexed by m, the transmitted intensity $I_{m}$ is related to the unattenuated intensity $I_{0}$ through the Beer–Lambert law. Taking the negative logarithm yields the line integral of the linear attenuation coefficient f(r) along the ray path $L_{m}$,

(1) $$y_{m}=-\ln\;\left(\frac{I_{m}}{I_{0}}\right)=\int_{L_{m}}f\left(r\right)\;dl,$$

where $y_{m}$ is the projection value at the m‐th detector cell, r denotes a position on the ray path $L_{m}$, f(r) is the value of the attenuation field at that position, and dl is the differential path length along the ray. This is known as the X‐ray transform and constitutes the forward model of the imaging system.

In practice, the continuous object function f(r) is represented on a discrete grid of N voxels with values $x_{n}$, and the projection is computed for M detector cells. The discrete forward projection can then be expressed as:

(2) $$y_{m}=\sum_{n=1}^{N}a_{mn}\;x_{n},\;m=1,\text{…},M,$$

where $a_{mn}$ represents the contribution of the n‐th voxel to the m‐th detector measurement. In matrix form, this corresponds to y=Ax, where $A\in R^{M\times N}$ is the system matrix.

Similarly, the backprojection operation distributes detector measurements back into the voxel domain:

(3) $$x_{n}=\sum_{m=1}^{M}b_{mn}\;y_{m},\;n=1,\text{…},N,$$

where $b_{mn}$ represents the contribution of the m‐th detector cell to the n‐th voxel.

The fundamental distinction among projection topologies lies in how the coefficients $a_{mn}$ and $b_{mn}$ are computed. This reflects how stages such as the geometric mapping, the interpolation scheme, and the integration procedure are formulated. The choices made at this level are decisive for both algorithmic design and implementation strategy, and they ultimately shape the accuracy and performance of the resulting method. The following subsection introduces a classification framework that systematically distinguishes these approaches at the levels of topology, algorithm, and implementation.

### 2.2 New classification

To systematically analyze and compare different approaches to projection and backprojection, we introduce a hierarchical classification framework consisting of three distinct but interrelated levels: *topology*, *algorithm*, and *implementation*. This framework helps to isolate conceptual design from computational strategies and low‐level code optimizations, thereby providing a clearer basis for understanding the strengths and limitations of existing methods.

#### 2.2.1 Topology

The *topology* level defines the geometric and structural description of the system. This includes how the X‐ray source, object, and detector are modeled, as well as how rays are traced through the volume. At this level, fundamental decisions are made about how the system is abstracted—for example, whether voxels are treated as points or bounded regions, whether rays are considered as center lines or fan‐shaped cones, and whether detectors are modeled as discrete points or as surfaces.

Topology fundamentally shapes the nature of the projection problem and places constraints on what algorithms can be developed under that system description. Once the topology is chosen, it sets the conceptual foundation for algorithm design.

#### 2.2.2 Algorithm

The *algorithm* level focuses on solving the projection or backprojection problem as defined by the topology. It addresses the *how*—how the information is transferred from voxel space to projection space and the vice versa. This includes techniques such as ray tracing, interpolation schemes, integral approximations, and spatial mappings. Algorithms can vary significantly even within the same topology, offering different trade‐offs in terms of accuracy and performance.

#### 2.2.3 Implementation

At the *implementation* level, the algorithm is realized in code and adapted to available computational resources. This level deals with concrete concerns such as programming language, memory layout, hardware acceleration, branching, and parallelization. While different implementations of the same algorithm may yield different execution times or memory usage, they operate under the same algorithmic logic.

We can reinterpret the commonly used topologies under this classification to better understand their design and evolution:

(1) **Pixel‐Driven (PD)** defines a topology where each voxel is treated as a point. The voxel center $p_{i,j,k}$ is geometrically mapped onto the detector plane to obtain a continuous coordinate:
(4) $$\xi =P_{S}\left(p_{i,j,k}\right),$$
  where $P_{S}$ denotes the geometric projection operator through the source point S onto the detector plane, and ξ is the resulting continuous position on the detector. The projection value corresponding to the voxel is then computed from discrete detector samples $y_{\ell }$ at positions $\xi_{\ell }$ using an interpolation kernel h,
(5) $$\hat{y}\left(\xi \right)=\sum_{\ell }h\left(\xi_{\ell }-\xi \right)\cdot y_{\ell },$$
  where $\xi_{\ell }$ are the discrete detector cell center positions and h is the interpolation kernel (e.g., linear or nearest‐neighbor). The primary algorithmic task in PD is therefore twofold: computing the geometric mapping in (4) and evaluating the interpolation in (5). Trigonometric formulations have been commonly employed for this mapping under standard parallel‐ and fan‐beam geometries.^3^ Due to its voxel‐centric nature, PD is computationally expensive and better suited for backprojection. Implementation challenges often revolve around managing memory access in nested voxel loops.
(2) **Ray‐Driven (RD)** models the detector as a grid of center points and traces rays from the source to each detector cell. The continuous projection value for a given ray is the line integral already introduced in (1). In the discrete case, this integral is approximated as a weighted sum of the voxel densities ρ(i,j,k), where the weights are the intersection lengths l(i,j,k) of the ray with each voxel,
(6) $$y_{m}=\sum_{i}\sum_{j}\sum_{k}l\left(i,j,k\right)\;\rho \left(i,j,k\right).$$
  Here ρ(i,j,k) is the voxel value (the $x_{n}$ of (2) in 3D indexing) and l(i,j,k) is the intersection length, which plays the role of the system coefficient $a_{mn}$. This form follows from treating f(r) as piecewise constant within each voxel. Unlike PD, where the weighting is based on interpolation, in RD the contribution of each voxel is determined by the physical intersection length of the ray with that voxel. The primary algorithmic task is therefore to identify which voxels each ray intersects and to compute the corresponding intersection lengths. Algorithms such as those by Siddon^6^ and Joseph^5^ address this task with different strategies: Siddon computes exact intersection lengths using parametric ray representations, while Joseph samples the volume along the ray using interpolation between neighboring voxels. More recent studies, such as those by Gao^9^ and Chen et al.,^8^ aim to compute this intersection‐based weighting more efficiently and exploit the parallelism of modern processors. RD is well‐aligned for forward projection but can skip voxels in backprojection, introducing artifacts. Implementation efficiency is often achieved through vectorization and ray‐parallel processing.
(3) **Distance‐Driven (DD)** defines both voxels and detector cells as bounded rectangular regions. The formulation is presented here in 2D for clarity; in 3D, the same procedure is applied separably along each axis. The boundaries of each pixel $\left[p_{n,L},\;p_{n,R}\right]$ and each detector cell $\left[d_{m,L},\;d_{m,R}\right]$ are mapped onto a common intermediate plane via a geometric mapping M, which projects the boundaries along the dominant axis toward the chosen common plane,
(7) $$p_{n}^{\prime }=M\left(p_{n,L},\;p_{n,R}\right),\;d_{m}^{\prime }=M\left(d_{m,L},\;d_{m,R}\right),$$
  where $p_{n}^{\prime }=\left[p_{n,L}^{\prime },\;p_{n,R}^{\prime }\right]$ and $d_{m}^{\prime }=\left[d_{m,L}^{\prime },\;d_{m,R}^{\prime }\right]$ are the mapped boundary intervals on the common plane, with the subscripts L and R denoting the left and right boundary coordinates of each pixel or cell. The contribution of each voxel to a detector cell is then determined by the overlap ratio,
(8) $$a_{mn}=\frac{\max\;\left(0,\;\min\left(p_{n,R}^{\prime },\;d_{m,R}^{\prime }\right)-\max\left(p_{n,L}^{\prime },\;d_{m,L}^{\prime }\right)\right)}{d_{m,R}^{\prime }-d_{m,L}^{\prime }}.$$
  The coefficient $a_{mn}$ in (8) is then used as the weighting factor in the general discrete projection formulation given in (2). This topology enables symmetric treatment of forward and backward projections and sequential memory access, but imposes strict geometric constraints. The main algorithmic challenge lies in accurately computing the overlapping areas, particularly in 3D, due to irregular intersections, distorted boundaries caused by tilted detector and source configurations, and index matching difficulties arising from the resulting irregular shapes, as discussed in Section 1.

By separating these levels, we can more effectively identify where the limitations of existing approaches lie—whether in the conceptual design (topology), mathematical formulation (algorithm), or computational realization (implementation). This perspective also motivates the development of the proposed VD topology, which seeks to simplify algorithm design while improving geometric generality and computational performance.

### 2.3 A new topology: vector‐driven

The vector‐driven (VD) topology introduces a unified and flexible framework for modeling forward and backward projection using vector algebra. Unlike traditional topologies that rely on geometric intersections or common mapping planes, VD directly models all system components as vectors in $R^{3}$. The key definitions are as follows.

The *detector plane* is defined by a reference origin point $O_{d}$ and two spanning vectors du and dv. Any point on the detector plane can be expressed as:

(9) $$r_{d}\left(s,t\right)=O_{d}+s\;\mathrm{du}+t\;\mathrm{dv},$$

where $O_{d}$ is the reference origin of the detector (corner of the first cell), du and dv are vectors describing the orientation and cell spacing along the detector columns and rows, and s and t are continuous coordinates on the detector plane. The center of the detector cell at discrete indices (u,v), denoted d(u,v), is obtained by evaluating (9) at integer s=u and t=v.

The *object volume* is defined by a reference origin $O_{o}$ and three spanning vectors $\hat{x}$, $\hat{y}$, and $\hat{z}$. Any point within the volume can be expressed as:

(10) $$r_{o}\left(x,y,z\right)=O_{o}+x\;\hat{x}+y\;\hat{y}+z\;\hat{z},$$

where $O_{o}$ is the reference origin of the object volume, $\hat{x}$, $\hat{y}$, $\hat{z}$ are the spanning vectors along each axis, and x, y, z are continuous coordinates. For a standard rectilinear voxel grid, the spanning vectors are aligned with the coordinate axes: $\hat{x}={\left(1,0,0\right)}^{T}$, $\hat{y}={\left(0,1,0\right)}^{T}$, and $\hat{z}={\left(0,0,1\right)}^{T}$, scaled by the voxel dimensions.

The *X‐ray source* is represented as a single point in 3D space:

(11) $$S={\left(S_{x},\;S_{y},\;S_{z}\right)}^{T}\in R^{3}.$$

By representing all system components in this unified vector form, both spatial positions and the directional relationships between them (such as rays or projections) share the same mathematical structure and can be subject to the same algebraic operations. This offers a more coherent framework than conventional trigonometric formulations, where spatial and angular computations are interleaved. Moreover, since vectors are naturally decomposable along axes and planes, the algorithms derived from this representation can be readily separated into independent operations, which facilitates efficient parallelization.

**FIGURE 3:**
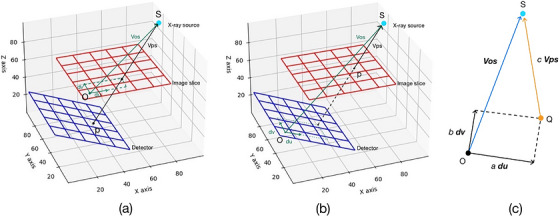
Illustration of the non‐orthogonal projection and vector‐based structure used in the VD topology. Projection operations are performed by projecting points onto target planes. (a): Forward projection, where detector cell centers are projected onto the image slice plane. (b): Backward projection, where voxel centers are projected onto the detector plane. (c): A system of linear equations is constructed using vector relationships to compute the projected positions in both forward and backward projections (the point Q represents the projection of point p onto the target plane). The symbols and their definitions are given in Table 1.

Additionally, the VD topology treats forward and backward projections symmetrically, since the same non‐orthogonal projection is applied in both directions. The only difference is which side acts as the destination and which side as the source plane, as detailed below. As illustrated in Figure 3, the VD topology operates by projecting points onto planes via non‐orthogonal projection, depending on the position of the source. In forward projection, each detector cell is treated as the destination, and the line integral that fills it is accumulated by mapping the cell onto every object slice in turn and reading the slice value at the projected position. In backprojection, each voxel is treated as the destination, and its value is obtained by mapping it onto the detector plane and reading the projection at the projected position. In both cases, the destination point drives the iteration, vector‐based calculations determine the projected location on the source plane, and interpolation provides the value at that position from neighbouring voxels or cells.

Thanks to this symmetry, the VD topology can describe both PD and RD within the same geometric schema. In forward projection, the vectors are cast as rays from detector cell centers toward the source and traced through the object slices, reproducing the RD integration pattern. In backward projection, they run from voxel centers toward the source and terminate on the detector plane modeled by Equation (9), mirroring the voxel‐iteration pattern of PD backprojection. VD therefore does not merely resemble PD and RD but contains them as instances of a single vector‐based description. Moreover, unlike PD or RD, it can be applied in both directions without the computational penalties or accuracy losses that each of them suffers in one direction. Compared to DD, VD achieves this unification without requiring an intermediate common plane, and thus avoids the distortions that DD exhibits under arbitrary rotations.

### 2.4 Algorithm and mathematical formulation

The algorithm must satisfy two criteria, consistency with the described topology and feasibility for implementation. Under VD, the projection problem reduces to two main tasks. The first is *non‐orthogonal projection*, which computes the position where a point (detector cell or voxel center) projects onto a target plane using vector projection principles. The second is *interpolation*, which estimates the value at the projected position from nearby values on the target plane.

#### 2.4.1 Non‐orthogonal projection

In the proposed approach, the projection of a point in three‐dimensional space onto a target plane is formulated using vector operations. As illustrated in Figure 3, this method defines all system components and their spatial relationships as vectors, enabling a consistent and unified structure for both forward and backward projections. The idea is to trace the direction of the ray emitted from the source point *S* through a given point p, and determine where this ray intersects the target plane. The plane is described by a reference point *O* and two spanning vectors, du and dv, which define its orientation in space. Rather than relying on classical geometry, the projected location is found by expressing the intersection point as a linear combination of the spanning vectors, yielding coordinates in the local frame of the plane.

This setup is general and symmetric, allowing the same mathematical framework to be applied in both directions of projection:

- In **forward projection**, the point p is a detector cell center, and the target plane is a slice from the 3D object. See Figure 3a
- In **backprojection**, the point p is a voxel center, and the plane is the detector surface. See Figure 3b

This symmetry eliminates the need for any intermediate mapping planes or specific adjustments for forward and backward projections.

As shown in Figure 3c, let $v_{\mathrm{OS}}$ denote the vector from the target‐plane reference point O to the source S, and $v_{\mathrm{PS}}$ the vector from the projected point p to the source S. The vector $v_{\mathrm{OS}}$ can then be expressed as a linear combination of du, dv, and $v_{\mathrm{PS}}$, as formulated in (12),

(12) $$v_{\mathrm{OS}}=a\mathrm{du}+b\mathrm{dv}+cv_{\mathrm{PS}}$$

where, the coefficients *a* and *b* represent the scaling factors of du and dv, respectively, while *c* is the scaling factor for the vector from the point to the source. To determine the projection of the point on the target plane, it is sufficient to compute *a* and *b*, while *c* is redundant. All vectors in (12) are known, and the coefficients can be obtained by solving the system of linear equations represented in a matrix form shown in (13).

(13) $$\left[\begin{array}{ccc} | & | & |\\ \mathrm{du} & \mathrm{dv} & v_{\mathrm{PS}}\\ | & | & | \end{array}\right]\left[\begin{array}{c} a\\ b\\ c \end{array}\right]=\left[\begin{array}{c} |\\ v_{\mathrm{OS}}\\ | \end{array}\right]$$

Some well‐known methods such as Gauss‐Jordan elimination in linear algebra can be used to find the solution. However, this approach is avoided because the operation must be repeated for all $v_{\mathrm{PS}}$ vectors in the system, which are numerous. Although the system of linear equations is limited to 3×3, the cumulative computational cost of such repetition becomes excessive. Additionally, the coefficient *c* is redundant, yet solving the entire system requires effort to compute it as well. Note that this inefficiency would arise during the implementation stage, but by recognizing it during the algorithm stage, we can reorient our approach to develop a more efficient solution.

To address this inefficiency, we extend our mathematical formulation further. In (12), the term containing the coefficient *c* should be eliminated, as it is redundant. To eliminate the term including *c*, we can apply cross product with $v_{\mathrm{PS}}$ to both sides, since the cross product of a vector itself results in zero. Thus, we obtain:

(14) $$v_{\mathrm{PS}}\times v_{\mathrm{OS}}=v_{\mathrm{PS}}\times \left(a\mathrm{du}\right)+v_{\mathrm{PS}}\times \left(b\mathrm{dv}\right)+v_{\mathrm{PS}}\times \left(cv_{\mathrm{PS}}\right)$$

(15) $$v_{\mathrm{PS}}\times v_{\mathrm{OS}}=a\left(v_{\mathrm{PS}}\times \mathrm{du}\right)+b\left(v_{\mathrm{PS}}\times \mathrm{dv}\right)+c(v_{\mathrm{PS}}\times v_{\mathrm{PS}}\text{APTARASTROKE})$$

(16) $$v_{\mathrm{PS}}\times v_{\mathrm{OS}}=a\left(v_{\mathrm{PS}}\times \mathrm{du}\right)+b\left(v_{\mathrm{PS}}\times \mathrm{dv}\right)$$

In (16), we reduced the vector equation to include only the coefficients *a* and *b*. Now, (16) needs to be solved for *a* and *b* individually. Using a similar method as in (14), we can apply the dot product to both sides of the equation to cancel out specific terms. We know that $\left(v_{\mathrm{PS}}\times \mathrm{dv}\right)$ is always orthogonal to dv, as the cross product of two vectors always produces a vector perpendicular to both operands. Additionally, the dot product of two orthogonal vectors is zero. Therefore, by taking the dot product of both sides with dv, we can eliminate the term that includes *b*:

(17) $$\mathrm{dv}\cdot \left(v_{\mathrm{PS}}\times v_{\mathrm{OS}}\right)=\mathrm{dv}\cdot a\left(v_{\mathrm{PS}}\times \mathrm{du}\right)+\mathrm{dv}\cdot b(v_{\mathrm{PS}}\times \mathrm{dv})\text{APTARASTROKE}$$

Now, both sides of the equation are scalars, as the dot product produces a single scalar. We can solve (17) to isolate *a*:

(18) $$a=\frac{\mathrm{dv}\cdot (v_{\mathrm{PS}}\times v_{\mathrm{OS}})}{\mathrm{dv}\cdot (v_{\mathrm{PS}}\times \mathrm{du})}$$

Similarly, to solve for *b*, we can apply the same reduction process to the vector equation. This time, by taking the dot product of both sides with du, we can eliminate the term that includes *a*:

(19) $$\mathrm{du}\cdot \left(v_{\mathrm{PS}}\times v_{\mathrm{OS}}\right)=\mathrm{du}\cdot a(v_{\mathrm{PS}}\times \mathrm{du})\text{APTARASTROKE}+\mathrm{du}\cdot b\left(v_{\mathrm{PS}}\times \mathrm{dv}\right)$$

This results in the scalar solution for *b*:

(20) $$b=\frac{\mathrm{du}\cdot (v_{\mathrm{PS}}\times v_{\mathrm{OS}})}{\mathrm{du}\cdot (v_{\mathrm{PS}}\times \mathrm{dv})}$$

Using (18) and (20), we determine the local coordinates (a,b) of the projection of the point.

Through this approach, we solve the linear equation system in (12) for *a* and *b* individually, without the need for inverse matrix operations in (13), making it computationally more efficient.

It is important to note that the operations performed in both the numerator and denominator of (18) and (20) involve a special operation called the *scalar triple product* which has a property known as the *circular shift property*, expressed as:

(21) A·(B×C)=B·(C×A)=C·(A×B)

where A, B, and C are arbitrary vectors in 3D. Note that the only term iterated during the projection process is $v_{\mathrm{PS}}$, while all other vectors remain constant for a single projection. Using this prior information, we can further optimize (18) and (20) to simplify the computational task during the implementation. Since the dot product is computationally much less expensive than the cross product, we can use this property to carry $v_{\mathrm{PS}}$ out of the parentheses to reduce the computation cost. Applying this property in (21) to both *a* and *b* in (18) and (20), we obtain:

(22) $$\begin{array}{ccc} a & =\frac{v_{\mathrm{PS}}\cdot \left(v_{\mathrm{OS}}\times \mathrm{dv}\right)}{v_{\mathrm{PS}}\cdot \left(\mathrm{du}\times \mathrm{dv}\right)}\;,\;b & =\frac{v_{\mathrm{PS}}\cdot \left(v_{\mathrm{OS}}\times \mathrm{du}\right)}{v_{\mathrm{PS}}\cdot \left(\mathrm{dv}\times \mathrm{du}\right)} \end{array}$$

In these forms of the equations, $\left(v_{\mathrm{OS}}\times \mathrm{dv}\right)$ and $\left(v_{\mathrm{OS}}\times \mathrm{du}\right)$ do not change during a single projection since they do not depend on the points being projected. Additionally, (du×dv) is the normal vector of the detector, which remains constant unless the detector is moved, and (dv×du) is the same vector with an opposite direction. Through the mathematical steps from (12) to (22), we have been able to efficiently solve the non‐orthogonal projection problem.

#### 2.4.2 Interpolation

After finding the local position of the projected point *(a*, *b)*, the corresponding value for this point needs to be calculated. This can be applied by interpolating neighboring points on the target plane. There are various methods for interpolation.^19^ In this work, we used slice‐wise bilinear interpolation, and the interpolated values across slices were subsequently accumulated for integration.

To perform this task efficiently, two main steps are required. First, the indices of the neighboring points on the target plane must be identified. These neighboring points correspond to the corners of the rectangular region that contains the projected point. Second, the proportional distances between the projected point and these neighboring points must be computed.

**FIGURE 4:**
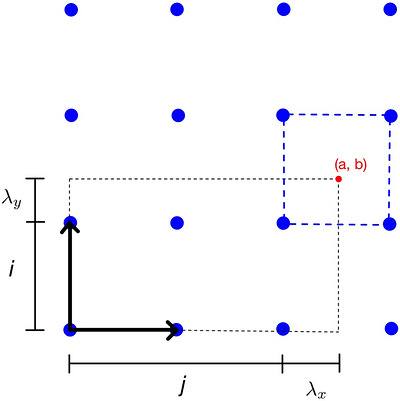
Illustration of how the index values (i, j) and the bilinear interpolation coefficients $\lambda_{x}$ and $\lambda_{y}$ are extracted from the projected point (a,b) directly. Blue dots represent the discrete sampling points on the target planevoxel centers in forward projection and detector cell centers in backward projection. The projected point (a,b) is interpolated using its four neighboring points to compute a continuous value via bilinear interpolation.

The values *a* and *b* in (22) can be directly used to perform both steps efficiently. As explained earlier, these values represent the scaling factors of the du and dv vectors. Each of these values consists of two components: an integer part and a fractional part. The integer part directly determines the index of the bottom‐left corner of the rectangular region containing the projected point, while the fractional part represents the proportional distance of the projected point from this corner as shown in Figure 4.

Mathematically, these components can be expressed as:

(23) $$i=\left\lfloor b\right\rfloor ,\;j=\left\lfloor a\right\rfloor ,\;\lambda_{x}=a-j,\;\lambda_{y}=b-i$$

where:

(1) i and j are the indices of the bottom‐left neighbor corner of the projected point. These are local indices within the 2×2 neighborhood, distinct from the voxel indices (i,j,k) used elsewhere.
(2) $\lambda_{x}$ and $\lambda_{y}$ represent the fractional distances of the projected point along the du and dv directions, respectively.
(3) ⌊·⌋ denotes the floor function, which extracts the integer part of a number.

This formulation allows for an efficient computation of the interpolation process without requiring iterative operations.

Based on (23), the interpolation for each projected point can be computed as:

(24) $$\begin{array}{cc} g(\lambda_{x},\lambda_{y}) & =\left(1-\lambda_{x}\right)\left(1-\lambda_{y}\right)\;g_{i,j}\\ & \;+\lambda_{x}\left(1-\lambda_{y}\right)\;g_{i,j+1}\\ & \;+\left(1-\lambda_{x}\right)\lambda_{y}\;g_{i+1,j}\\ & \;+\lambda_{x}\lambda_{y}\;g_{i+1,j+1}, \end{array}$$

where $g_{i,j}$ is the value at the (i,j) corner of the 2×2 neighborhood and $g(\lambda_{x},\lambda_{y})$ is the interpolated value at fractional offsets $\left(\lambda_{x},\lambda_{y}\right)$ within it. In this work, g represents the voxel values on a target slice (forward projection) or the pre‐normalized detector measurements (backward projection).

Using this formulation, both indices and the interpolated values can be computed with simple arithmetic operations without requiring iterative procedures. The interpolation itself is the standard bilinear scheme, applied here to the coefficients (a,b) obtained from (22).

### 2.5 Implementation

The pseudo‐codes in Algorithms 1 and 2 outline the implementation procedures for forward and backward projections, respectively. In the forward projection, the detector‐level precomputation pass and the per‐slice detector traversal are both parallelizable over the detector indices (u,v), with the inner sweeps reusing the stored per‐cell quantities through cheap affine updates. In the backward projection, the outer (i,j) iterations of the voxel loop are likewise parallelizable, and the inner voxel‐column loop reduces to a few additions and two divisions per voxel, thanks to the row‐level partial sums kept out of the innermost loop.

The spanning vectors du and dv in (22) refer to the target plane of the current projection direction. In backward projection, the target plane is the physical detector, with spanning vectors defined in (9). In forward projection, the target plane is the image slice, whose spanning vectors are $\Delta_{x}\;\hat{x}$ and $\Delta_{y}\;\hat{y}$, where $\Delta_{x}$, $\Delta_{y}$, $\Delta_{z}$ denote the voxel spacings along the x, y, z axes (Table 2).

**TABLE 1:** Definitions and acronyms of key variables.

Symbol	Definition
***O***	Reference point on the target plane, serving as the local origin for the projection coordinate system.
***S***	Position of the X‐ray source.
p	The 3D point to be projected onto the target plane.
du	The vector defining the horizontal axis (local *x*‐direction) of the target plane; its magnitude equals the detector cell width.
dv	The vector defining the vertical axis (local *y*‐direction) of the target plane; its magnitude equals the detector cell height.
$v_{\mathrm{OS}}$	Vector from the target plane origin *O* to the source *S*.
$v_{\mathrm{PS}}$	Vector from the point p to the source *S*.

**TABLE 2:** Acquisition geometry.

Metric	Value	Unit
Source–isocenter distance (SID)	700	mm
Source–detector distance (SDD)	900	mm
Object–detector distance (ODD)	200	mm
Detector elements $N_{v}\times N_{u}$	800×800	—
Detector element size $\Delta_{v}\times \Delta_{u}$	0.25×0.25	mm^2^
Detector active area $N_{v}\;\Delta_{v}\times N_{u}\;\Delta_{u}$	200×200	mm^2^
Object dimensions (voxels) $N_{z}\times N_{y}\times N_{x}$	1×400×400	—
Voxel size $\Delta_{z}\times \Delta_{y}\times \Delta_{x}$	0.25×0.25×0.25	mm^3^
Volume extent (height × depth × width)	0.25×100×100	mm^3^

The per‐cell quantities are the base coefficients $\left(a_{0},b_{0}\right)$, the per‐slice shifts (Δa,Δb), and the normalization coefficient $c_{uv}$. The base coefficients are the values of (a,b) from (22) evaluated on the first slice (i=0) for each detector cell. The shifts are the per‐voxel changes in (a,b) along the slice axis, constant across slices for each cell. The coefficient $c_{uv}$ is the denominator of (22) evaluated at detector cell d(u,v). Since this denominator depends only on $v_{\mathrm{PS}}$, du, and dv, which are all constant across the inner iterations, it is precomputed once per cell.

The affine decomposition used in Algorithm 2 follows from (22): with the voxel center at index (i,j,k) written as $O_{o}+i\;\Delta_{z}\;\hat{z}+j\;\Delta_{y}\;\hat{y}+k\;\Delta_{x}\;\hat{x}$, the numerators $N_{a}$, $N_{b}$ and the denominator D are affine in (i,j,k). Their values at the reference voxel (0,0,0) are denoted $A_{0}$, $B_{0}$, $D_{0}$, and their per‐axis increments by the triples $\left\{A_{x},A_{y},A_{z}\right\}$, $\left\{B_{x},B_{y},B_{z}\right\}$, $\left\{D_{x},D_{y},D_{z}\right\}$, where, for example, $A_{x}$ is the change in $N_{a}$ when advancing one voxel along the x axis. In Algorithm 2, i indexes slices along z, j voxel rows along y, and k voxel columns along x.

ALGORITHM 1 **Forward Projection**

**Table:** 

1:	Initialize $v_{\mathrm{PS}}$, $v_{\mathrm{OS}}$, together with the extension vectors du, dv of the detector and those of an image slice.
2:	Project object corners onto the detector plane via (22) with the detector's du, dv, and obtain the clipping region $\left[v_{\min},v_{\max}\right]\times \left[u_{\min},u_{\max}\right]$.
3:	**for** each detector row v in the clipping region **do**
4:	**for** each detector column u in the clipping region **do**
5:	Evaluate $\left(a_{0},b_{0}\right)$ from (22) with the slice's du, dv on the first slice, and precompute the per‐slice shifts (Δa,Δb) and the normalization coefficient $c_{uv}$.
6:	**end for**
7:	**end for**
8:	**for** each slice i in the object **do**
9:	**for** each detector row v in the clipping region **do**
10:	**for** each detector column u in the clipping region **do**
11:	$a\leftarrow a_{0}+i\;\Delta a$; $b\leftarrow b_{0}+i\;\Delta b$.
12:	Obtain indices and fractions $\left(\lambda_{x},\lambda_{y}\right)$ from (23).
13:	Accumulate the bilinear contribution (24), scaled by $c_{uv}$, into detector cell (u,v).
14:	**end for**
15:	**end for**
16:	**end for**

ALGORITHM 2 **Backprojection**

**Table:** 

1:	Initialize constants $v_{\mathrm{PS}}$, $v_{\mathrm{OS}}$, du, dv.
2:	Express $N_{a}$, $N_{b}$, D in (22) as affine function of (i,j,k), for example, $N_{a}\left(i,j,k\right)=A_{0}-iA_{z}-jA_{y}-kA_{x}$, and analogously for $N_{b}$ and D. Precompute the bases $A_{0},B_{0},D_{0}$ and the strides $\left\{A_{x},A_{y},A_{z}\right\}$, $\left\{B_{x},B_{y},B_{z}\right\}$, $\left\{D_{x},D_{y},D_{z}\right\}$.
3:	**for** each detector row v **do**
4:	**for** each detector column u **do**
5:	Precompute the normalization coefficient $c_{uv}$ and form the pre‐normalized projection $\tilde{y}\left(u,v\right)\leftarrow c_{uv}\;y\left(u,v\right)$ in place.
6:	**end for**
7:	**end for**
8:	**for** each slice i **do**
9:	**for** each voxel row j **do**
10:	$D^{ij}\leftarrow D_{0}-iD_{z}-jD_{y}$; $N_{a}^{ij}\leftarrow A_{0}-iA_{z}-jA_{y}$; $N_{b}^{ij}\leftarrow B_{0}-iB_{z}-jB_{y}$.
11:	**for** each voxel column k **do**
12:	$D\leftarrow D^{ij}-kD_{x}$; $a\leftarrow (N_{a}^{ij}-kA_{x})/D$; $b\leftarrow (N_{b}^{ij}-kB_{x})/D$.
13:	Obtain indices and fractions $\left(\lambda_{x},\lambda_{y}\right)$ from (23).
14:	Update voxel (i,j,k) via the bilinear interpolation (24).
15:	**end for**
16:	**end for**
17:	**end for**

In the implementation stage, the algorithm designed in the previous subsection will be evaluated based on two key aspects: *computation efficiency* and *memory utilization*.

#### 2.5.1 Computation efficiency

Computation efficiency can be evaluated by analyzing the nested loop structure and the dependencies of the parameters in the implementation. The algorithm inherently requires a deeply nested loop structure. However, if the parameters are well‐organized and the dependencies between the parameters and iterations are sufficiently minimized, the problem can be optimized using both vectorization and parallelization.

In (22), we derived the projection of each point based solely on the terms related to the point *(a*, *b)* and constant terms for a single projection, such as $v_{\mathrm{PS}}$, $v_{\mathrm{OS}}$, du, and dv. Furthermore, in (23), we determined the indices of the corresponding rectangular region on the target plane for the projected point without requiring sequential computations, such as iterations or increments. Thanks to these improvements, the inner loops for computing detector values are easily vectorizable and parallelizable, with a regular 2D access pattern that maps cleanly onto SIMD lanes and the texture caches of modern GPUs.

#### 2.5.2 Memory utilization

Memory access patterns are another critical aspect of evaluating the efficiency of an implementation. When values are distributed across random locations in memory, cache optimization becomes difficult for the processor, increasing the data transfer load. Conversely, if accessed values are well‐organized and stored close to each other, data transfer between different memory levels is significantly improved.

The proposed implementation not only improves computational efficiency but also enhances memory management. Unlike traditional RD algorithms such as Siddon's algorithm,^6^ which often require accessing large portions of the volume simultaneously, this approach computes projections on a per‐voxel basis by projecting each point directly onto the plane. This localized access pattern reduces the need to load the entire volume into memory at once, thereby decreasing cache misses and minimizing performance degradation caused by irregular or scattered data access. Furthermore, because the non‐orthogonal projection and index calculations are independent of neighboring iterations, the overall computation can be easily divided into smaller parts, facilitating operations under high memory demands.

## 3 RESULTS

Based on the methodology described above, various experiments are conducted to evaluate and compare the efficiency of the proposed method. The evaluation focuses on two main aspects: *Accuracy* and *Computational Performance*. Accuracy refers to the quality of the forward and backward projections, including the artifact behavior of each topology under different geometrical conditions. Computational performance, on the other hand, is assessed across multiple hardware‐level metrics such as execution time, the number of floating‐point operations (GFLOP per call), instructions per cycle (IPC), and DRAM read/write traffic, providing a comprehensive characterisation of the algorithms.

### 3.1 Experimental setup

Experiments are conducted using an X‐ray imaging simulation tool developed in C++. This tool provides highly configurable system parameters to mimic or modify a CT system. The acquisition parameters used in the system—such as the dimensions, spatial resolution (in mm), and relative distances are provided in Table 2. This initial configuration is used in the trials described in the following subsections, where different rotation scenarios are applied.

The first experiment, designed to evaluate accuracy, is conducted using a thin object consisting of a single slice in 3D Cartesian space. This configuration is deliberately chosen to eliminate the overlapping effects that inherently arise when projecting multiple slices in 3D volumes, thereby allowing the isolated impact of the projection and backprojection operators to be observed more clearly.

In selecting algorithms for each topology, two strategies were adopted to ensure a fair comparison in terms of both accuracy and computational performance. For accuracy evaluation, fundamental and widely accepted baseline algorithms were chosen to represent each topology in their most established form. Specifically, Siddon's algorithm^6^ was used for RD. For PD, two baselines were employed. The first, referred to as PD‐Basic, is an optimised voxel‐iteration implementation adapted from the TIGRE toolbox.^20^ The second is the SF‐TR algorithm.^17^ For DD, De Man's algorithm^11^ was used. For computational performance evaluation, the same algorithms were applied to larger and more demanding dimensions to better highlight differences in execution time and scalability.

### 3.2 Accuracy

**FIGURE 5:**
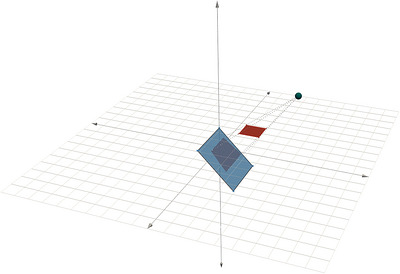
Experimental setup: the blue rectangle represents the detector plane, the small sphere is the X‐ray source, and the red rectangular plane is a thin object consisting of a single slice.

The accuracy of forward and backward projection is evaluated in terms of artifacts introduced by each method. To clearly examine these artifacts, we set up an acquisition geometry given in 2, first. Then, the source and detector are rotated isocentrically around the center of the object, with the rotation axis aligned to the y‐axis, as illustrated in Figure 5. Additionally, forward and backward projections are evaluated independently to avoid mixing artifacts from the projection stage when analyzing the backprojection results.

**FIGURE 6:**
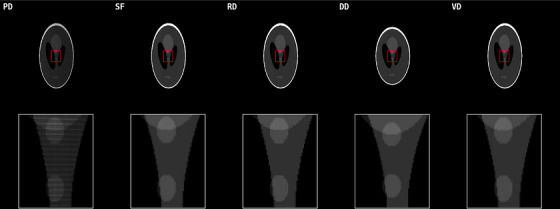
Projection results with a 45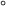 isocentric rotation of the source and detector around the *y*‐axis (i.e., pitch rotation).

Figure 6 shows the projections of a single‐slice 3D object, modeled using the Shepp–Logan phantom, onto the detector. In each image, horizontal compression is visible due to rotation around the *y*‐axis. Artifacts are clearly observed in the PD basic method, since iterating over voxel centers skips detector cells in between, and the missed cells receive no contribution when each voxel scatters its value onto the detector, leaving them as visible stripes. The other projection methods yield significantly better results. The DD method, however, exhibits a slightly different appearance compared to the others, showing an egg‐shaped distortion, although it does not present artifacts such as the Moiré pattern.

**FIGURE 7:**
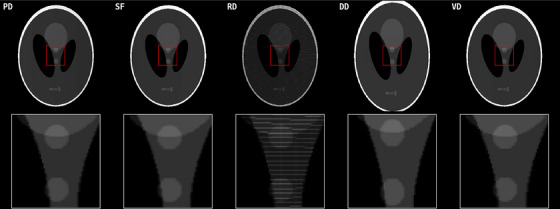
Backprojection results with a 45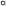 isocentric rotation of the source and detector around the *y*‐axis (i.e., pitch rotation).

In Figure 7, the backprojection results of the same phantom are presented. To eliminate artifacts arising from projection modeling and to ensure standardized initial conditions, the RD topology with the Siddon algorithm was employed for the forward projection (Figure 6). This setup isolates the effects attributable solely to the backprojection process. As shown in Figure 7, RD backprojection exhibits severe artifacts in a similar form, since iterating over rays skips voxels between adjacent rays, and the missed voxels receive no contribution when each ray scatters its value into the volume, leaving them as stripes in the reconstructed slice. The other methods provide more accurate backprojection. A similar egg‐shaped distortion and magnification issue is also observed in the DD backprojection.

**FIGURE 8:**
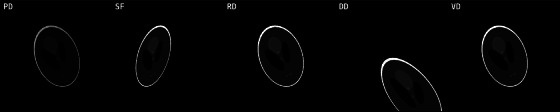
Projection results with a 30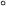 isocentric rotation of the source and detector around the both *y* and *z*‐axes.

To further evaluate the geometric robustness of the topologies under more challenging conditions, an isocentric rotation of the source and detector by 30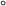 around both the *y*‐ and *z*‐axes was applied. As shown in Figure 8, the DD projection exhibits a pronounced spatial shift. This artifact results from the *index matching problem*, due to the index counting mechanism in DD, which assumes a regular geometry. Any algorithmic modification aimed at correcting this geometric inconsistency would inevitably bring a substantial computational cost, and the resulting irregular index access would break the sequential memory access pattern that constitutes the principal advantage of the DD approach^10^. In this configuration, the SF projection is tilted in the opposite direction relative to the other methods. The original SF formulation is derived under a circular orbit in which the detector axes are aligned with the transaxial plane and the rotation axis,^17^ and the SF relies on this alignment. Once a second rotation is added, this assumption is violated and a geometric tilt appears in the projection.

We can summarize the observations as follows:

(1) RD performs well in forward projection but causes artifacts in backprojection.
(2) Basic PD performs well in backprojection but introduces artifacts in forward projection.
(3) The SF method mitigates the artifacts of basic PD under single‐axis rotation. Under combined two‐axis rotation, however, the alignment between the detector axes and the rotation geometry assumed by the separable formulation^17^ no longer holds, and the resulting projections exhibit unavoidable geometric distortions.
(4) The DD method performs well in both forward and backward projections, but it is sensitive to challenging geometric conditions.
(5) VD produces artifact‐free output across all tested scenarios, with near‐perfect image‐quality scores matching the leading combination.

Based on this, a combination of forward and backward projection methods can be selected to achieve high‐quality reconstruction. For example, using RD for forward projection and PD for backprojection yields both rotation robustness and artifact resilience.^21^ To test such projector–backprojector combinations, we conducted a new experiment that pairs forward and backward operators across topologies. The setup uses 45‐degree rotations around the *y*‐axis only.

**FIGURE 9:**
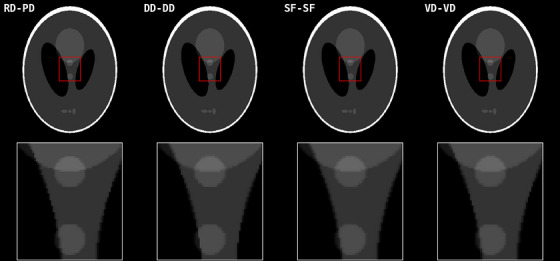
Hybrid forward and backward projection results for a 45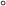 isocentric rotation of the source and detector around the *y*‐axis.

The projections are normalized before backprojection by computing normalization coefficients from a forward projection of an image containing only ones. This ensures that the backprojected image remains on the same scale as the original and is not affected by angle‐dependent differences caused by the point source. No additional filters are applied to enhance the image quality. As seen in Figure 9, the forward and backward projection results match closely to the original image.

Table 3 presents image quality metrics for quantitative comparison. According to this result, all four methods reach near‐perfect scores, and the small remaining differences place VD second across every metric behind the leading combination. The DD artefacts seen in the single‐direction tests are largely cancelled when DD is used in both directions, since the same geometric distortion appears in the forward and backward passes. This is the reason why round‐trip metrics alone cannot fully reveal a topology's directional behaviour, and why the projection and backprojection operators in this study are evaluated independently in the single‐direction experiments.

**TABLE 3:** Comparison of projector–backprojector combinations using image quality metrics.

Methods (forward ‐ backward)	SSIM	RMSE	PSNR	NCC	UIQI
RD (Siddon) ‐ PD (Basic)	0.9915	0.0276	31.1746	0.9918	0.9914
DD (De Man) ‐ DD (De Man)	0.9894	0.0307	30.2499	0.9899	0.9893
PD (SF‐TR) ‐ PD (SF‐TR)	0.9873	0.0336	29.4827	0.9880	0.9872
VD (Proposed) ‐ VD (Proposed)	0.9903	0.0294	30.6352	0.9908	0.9902

More broadly, such directional artifacts may interact non‐trivially with iterative reconstruction. Within such a loop, the backward operator is typically applied one more time than the forward operator, since the measured projections enter the loop directly. The residuals computed between simulated forward projections and the measured data are themselves shaped by the directional distortion of the forward operator and propagate into the subsequent updates. How such artifacts propagate through iterative reconstruction has not been systematically characterised, to our knowledge, and remains an open question for future work.

### 3.3 Computational performance

Alongside accuracy, computational performance is another essential criterion for comparing projection methods. Unlike accuracy, however, timing behavior reflects the joint effect of the algorithm, its implementation, and the underlying hardware. To observe computational performances of the projection and backprojection methods we report five complementary metrics and describe the measurement protocol below.

#### 3.3.1 Experimental setup

All experiments were conducted on a laptop running Ubuntu 24.04 LTS, equipped with an Intel Core i7‐12700H processor (12th generation, Alder Lake hybrid architecture with 6 performance and 8 efficient cores) and 64 GB of DDR4 memory. The code was written entirely in C++17 and parallelized with OpenMP, and compiled with GCC 13.3 using the flags ‐O3 ‐march = native ‐ffast‐math. No GPU acceleration or external compiled libraries were employed.

To ensure reproducibility, the execution environment was strictly controlled. Simultaneous multithreading (Intel Hyper‐Threading) was disabled to maintain a one‐to‐one mapping between logical threads and physical cores. Execution was constrained to the six performance cores (P‐cores), ensuring that the OpenMP workload was distributed across dedicated physical hardware without interference. To eliminate frequency scaling artifacts, the CPU governor was set to *performance* (rather than the default *powersave*) to ensure a consistent clock speed, and Intel Turbo Boost was disabled, locking all P‐cores to their nominal base frequency of 2.3 GHz. This fixed‐frequency approach prevents thermal throttling and dynamic clock adjustments, providing a stable baseline for hardware‐counter measurements.

The test configuration utilizes a cubic phantom of $1000^{3}$ voxels with an edge length of 200 mm, and a 1000×1000 element detector with a total area of 300×300 mm^2^. The source, object center, and detector center are aligned along a common optical axis, providing a maximum magnification of 1.33. No rotation is applied during the benchmark. At this scale, the working set substantially exceeds the 24 MB shared last‐level cache. Consequently, the memory hierarchy is stressed realistically, preventing the results from being biased by artificial cache residency and ensuring a more representative performance evaluation of the memory subsystem.

#### 3.3.2 Measured metrics and methodology

We report five metrics covering end‐to‐end cost, computational workload, pipeline efficiency, and main‐memory traffic. All five are collected in a single perf stat session from the Alder Lake P‐core PMU and the uncore memory‐controller counters.

(1) **Wall time (median, s)**. End‐to‐end runtime of a single call. Each algorithm runs once as a warm‐up, so that first‐touch timing anomalies do not enter the measurement. We then perform n=10 timed calls and report the median.
(2) **GFLOP per call**. Floating‐point operations per call, in billions. This is a purely algorithmic quantity. It reflects the arithmetic cost of producing the output and is independent of memory hierarchy or instruction scheduling. A lower value therefore means strictly fewer operations per invocation.
(3) **Instructions per cycle (IPC)**. Average retired instructions per CPU clock cycle, aggregated across the six P‐cores. IPC captures how effectively the pipeline is utilised. A higher value indicates fewer stalls from cache or memory latencies, branch mispredictions, and load/store dependencies. Two algorithms with similar FLOP counts can therefore differ in IPC when one has better data locality or more predictable control flow.
(4) **DRAM read per call (GB)**. Bytes read from DRAM during a single call, taken from the memory‐controller counters. The count includes both demand cache misses and hardware prefetches, and therefore reflects the full main‐memory read load generated during execution.
(5) **DRAM write per call (GB)**. Bytes written to DRAM during the call, from the same memory‐controller counters. Together with DRAM Rd, it characterises the full main‐memory access load produced by a single projection or backprojection call.

Each kernel call is isolated using perf stat gating, so that only the projection or backprojection time enters the measurement window. Background DRAM traffic is additionally sampled in a 5 s idle window before and after each call, and the measured values are corrected with this baseline so the reported values reflect only the algorithm's own main‐memory activity.

#### 3.3.3 Observations

Table 4 reports the five metrics for every projector and backprojector.

**TABLE 4:** Projection and backprojection performance at the $1000^{3}$ phantom / 1000×1000 detector configuration.

Projection	GFLOP/call	IPC	DRAM Rd (GB)	DRAM Wr (GB)	Time (s)
PD – Basic	29.05	3.64	5.65	0.28	1.623
PD – SF‐TR	26.38	1.51	69.58	0.16	5.087
RD – Siddon	16.92	2.34	7.49	0.01	5.696
DD – De Man	52.73	4.83	30.30	17.79	2.372
VD – Proposed	13.68	4.42	5.05	0.04	1.026
**Backprojection**	**GFLOP/call**	**IPC**	**DRAM Rd (GB)**	**DRAM Wr (GB)**	**Time (s)**
PD – Basic	27.04	3.45	5.25	4.03	1.603
PD – SF‐TR	91.71	1.79	69.83	52.47	12.882
RD – Siddon	8.84	2.16	4.65	1.71	4.120
DD – De Man	52.92	4.78	28.02	18.64	2.279
VD – Proposed	24.04	3.57	5.24	4.03	1.570

GFLOP/call reports the algorithmic workload, IPC the pipeline efficiency of the CPU, DRAM Rd and DRAM Wr the main‐memory read and write traffic per call, and Time the median wall‐clock duration of a single call.

In forward projection, VD achieves the lowest median wall time at 1.026 s. Its floating‐point workload (13.68 GFLOP per call) is also the lowest among the evaluated projectors. The IPC of 4.42 ranks second to DD–De Man's 4.83, yet VD reaches this level while performing roughly 4× less arithmetic. The DRAM read traffic of 5.05 GB stays close to the ∼4 GB nominal volume size, indicating that VD reads each voxel essentially once without extra main‐memory accesses. The write traffic, at 0.04 GB, is very small in relative terms. This is the expected behaviour for forward projection, where only the detector is written.

In backprojection, VD is again the fastest (1.570 s) but leads PD–Basic only narrowly (1.603 s). The PD baseline used here incorporates the same row‐level restructuring employed in production toolchains, ensuring a fair comparison. VD's small advantage comes from a slightly lower FLOP count (24.04 vs. 27.04) and a marginally higher IPC (3.57 vs. 3.45). The DRAM figures of the two methods coincide almost exactly at ∼5.2 GB read and ∼4.0 GB written, close to the practical floor set by the ∼4 GB reconstruction volume. With both methods already at this memory floor, the remaining difference reduces to arithmetic efficiency.

Among the remaining methods, DD–De Man is designed around sequential memory access. In our trials, however, the DRAM write traffic reaches ∼18 GB in both directions, reflecting the cost of building and updating the per‐slice boundary maps, and this write cost shapes the overall timing more than the sequential‐access advantage does. RD–Siddon keeps a low FLOP count (16.92 for projection, 8.84 for backprojection) through its selective ray traversal, yet its IPC stays below 2.4, so the irregular access pattern limits the achievable throughput. PD–SF‐TR carries a moderate arithmetic workload, yet its wall time stays well above the VD and PD–Basic baselines. The limiting factor is main‐memory traffic rather than arithmetic cost. The DRAM read volume is roughly an order of magnitude larger than the volume size, and backprojection adds a comparable write load because each voxel update follows a non‐contiguous indexing pattern set by the separable column traversal. The IPC remains low, consistent with a memory‐bound regime.

Overall, VD emerges as the most efficient approach for both forward and backward directions. Its performance advantage stems not from the dominance of a single metric, but from a balanced profile across computational workload, memory traffic, and pipeline efficiency. This profile is a direct consequence of the method's underlying topological restructuring rather than of any specific implementation‐level optimisation.

## 4 DISCUSSION

This study introduced a new hierarchical classification of the projection problem, structured across three interrelated levels: *topology*, *algorithm*, and *implementation*. This framework helps clarify the role of each component in the projection process and supports a more systematic foundation for analyzing and developing reconstruction methods.

Within this framework, we proposed the VD method, which combines a vector‐based topology, the derived projection and backprojection algorithms, and corresponding implementations built around linear vector operations. Unlike approaches that address only a single layer (e.g., algorithmic or implementation‐level refinements), VD is specified consistently across all three.

Rather than committing to a voxel‐based or cell‐based handling, VD describes the system as the non‐orthogonal projection of a point onto a plane. This unified description allows both cell‐based ray‐tracing and voxel‐based mapping operations to be derived within the same mathematical scheme. PD and RD algorithms emerge as particular solutions of the underlying equation, and even DD can be constructed by using voxel and detector cell corners in place of their centers. The TIGRE PD baseline used in this study is one such instance, corresponding to the special case in which (12) is solved for the scalar c along $v_{\mathrm{PS}}$. Because the VD description is built on relative position vectors, it can host established topologies as particular reductions within a single framework.

The experimental results confirm that the proposed method performs consistently in both forward and backward projections. These improvements arise from several key structural features:

(1) **Symmetry**: The same mathematical formulation applies to both projection directions, avoiding the artifacts seen in PD forward projection and RD backward projection.
(2) **Geometric robustness**: The method avoids intermediate mapping planes required in DD, and therefore does not exhibit the distortion, irregular intersections, or index mismatches that DD shows under complex orientations.
(3) **Computational efficiency**: The method reduces to compact vector operations. This yields a light arithmetic workload, efficient pipeline utilisation, and a relatively light memory‐access load. Together these properties make the method practical on modern multi‐core hardware.

Overall, the VD method avoids the characteristic artifacts inherent in the other topologies. It exhibits neither the directional asymmetry typical of PD and RD, nor the shape distortions and geometric fragility associated with DD. VD achieves these benefits while avoiding the computational overhead that SF incurs to improve pixel‐driven accuracy.

The implementation operates on localised, per‐point computations, so the full volume never needs to be resident in memory. Output elements are updated once and in order, which keeps access patterns sequential and friendly to hardware prefetch. The same independence allows the work to be partitioned into smaller blocks, which is useful in memory‐constrained settings. Many prior efforts optimise a single metric within an established method, such as GPU parallelisation or sequential memory access. Introducing a new topology and designing the algorithm and implementation coherently around it instead makes room for a balanced profile across arithmetic work, access pattern, and IPC. Individual methods may still surpass VD on any one metric, but none combines the three as evenly.

The algorithm is inherently parallel and predominantly branchless, with a single *if–else* guard used to skip unnecessary operations in the main loop. The per‐element operations are independent and amenable to vectorisation. These properties should carry over cleanly to GPU architectures, although a dedicated GPU implementation is left for future work.

Computational performance depends strongly on hardware capabilities and implementation details, which makes timing results difficult to generalise across studies. The present evaluation already moves beyond wall time alone. It reports floating‐point throughput, instruction‐level throughput, and DRAM read/write traffic in parallel, so the differences between algorithms can be traced to concrete architectural causes rather than to a single aggregate number. This multi‐metric view makes the sources of the observed speed‐up directly observable rather than concealing them behind total runtime.

A broader high‐performance‐computing characterisation is a natural extension of this analysis. Complementary metrics such as cache locality at each cache level, memory‐bandwidth saturation, and threading behaviour would refine the picture further. The same methodology could then be applied across a wider range of detector and object resolutions, as well as across CPU and GPU architectures.

The accuracy experiments are carried out on a single‐slice phantom rather than on a fully filled volume. The geometry itself remains three‐dimensional throughout, since the source, the detector, and the rotations are placed and exercised in 3D space, and the simplification concerns only the object. With a multi‐slice volume, the projections of the individual slices superpose on the detector, and the characteristic artefacts of each topology can be partially washed out by this superposition. The artefacts studied here are most informative when they are directly readable by eye, and the single‐slice setup keeps them visible without giving the proposed method any masking advantage either. The choice also leaves the quantitative evaluation on a clean footing. A forward projection followed by its backprojection returns to the same domain as the input, so the standard 2D image‐quality metrics in Table 3 apply without an intermediate reconstruction step. Reconstructing a multi‐slice volume with a method such as SART or FDK would otherwise mix the behaviour of the projection and backprojection operators with that of the outer reconstruction algorithm and its regularisation, and the reported numbers would no longer reflect properties of the operators alone.

This study focuses on cone‐beam scan geometry. The proposed method can be adapted to parallel‐beam configurations by replacing the point source with a fixed direction vector in the non‐orthogonal projection step. Similarly, while the VD topology is developed here for flat detector surfaces, it can be extended to curved detectors by adapting the surface model, or by transforming the detector into an equivalent flat representation.

In standard CT imaging, an isocentric rotation around a single axis is typically sufficient for acquisition. If the detector lies parallel to the xy‐plane, rotation around the *z*‐axis alone does not affect reconstruction, as the source and detector maintain the same facing direction. Consequently, more complex geometries may initially seem unnecessary. However, rotations around multiple or custom axes can enhance the reconstruction. Therefore, supporting flexible projection trajectories remains important for future 3D imaging systems.

The forward and backward algorithms presented here are not algebraic adjoints of each other. This is an algorithm‐level choice rather than a limitation of the VD topology itself, since a matched variant can be derived within the same topology as an extension aimed at iterative reconstruction. Moreover, the use of unmatched projector–backprojector pairs in iterative reconstruction is already an established setting whose convergence behaviour has been characterised.^21^ The present study evaluates the projection and backprojection operators in isolation, focusing on their artifact behaviour and computational cost.

The hierarchical classification introduced in this study offers a valuable tool for reinterpreting the existing literature. Many previous works mix or conflate the roles of topology, algorithm, and implementation, making it difficult to identify the source of observed limitations. Reorganizing the literature through this structured lens could improve clarity, facilitate fairer comparisons, and inspire new approaches grounded in a clearer understanding of system design.

## 5 CONCLUSION

This study introduced a three‐level distinction between topology, algorithm, and implementation, with contributions at each level. The topology is VD, a new projection method based on vector relations between voxels, source, and detector cells. The algorithm is derived from vector algebra and built around compact primitives such as dot and cross products. The implementation is tailored to 3D cone‐beam CT geometries. Together, these define a unified mathematical framework in which forward and backward projections share the same formulation and remain valid under arbitrary detector orientations and rotation axes.

In the geometric configurations tested, the VD operators produced artifact‐free output and matched the leading baseline on standard image‐quality metrics. They simultaneously attained the shortest wall‐clock time in both directions, with a balanced profile of low arithmetic workload, high pipeline efficiency, and low DRAM traffic. Taken together, these results show that VD avoids the directional artifacts and geometric fragility that limit established topologies, and that it does so without the computational penalty typically associated with more complicated projection methods like SF.

## FUNDING INFORMATION

This work was supported by the Scientific and Technological Research Council of Türkiye (TÜBİTAK) under Grant No. 1001‐123E028.

## CONFLICT OF INTEREST STATEMENT

The authors declare that a patent application related to the methods described in this work has been filed.

## Data Availability

No new datasets were generated during this study; all reported results were obtained from the numerical phantoms and simulated acquisitions described in the manuscript. The source code implementing the proposed method is not publicly available because it is the subject of a pending patent application, but further details may be obtained from the corresponding author upon reasonable request.
